# Correction: Expanding the role of village malaria workers in Cambodia: Implementation and evaluation of four health education packages

**DOI:** 10.1371/journal.pone.0315636

**Published:** 2024-12-09

**Authors:** Mipharny Betrian, Dafne Umans, Moul Vanna, Sam Ol, Bipin Adhikari, Chan Davoeung, James J. Callery, Yok Sovann, Thomas J. Peto, Richard J. Maude, Rob W. van der Pluijm, Voeurng Bunreth, Martin P. Grobusch, Michèle van Vugt, Yoel Lubell, Lorenz von Seidlein, Arjen M. Dondorp, Siv Sovannaroth, Dysoley Lek, Rupam Tripura

The 12^th^ author’s name is spelled incorrectly. The correct name is: Voeurng Bunreth.
