# Supplementary material for: Expanding the role of village malaria workers in Cambodia: Implementation and evaluation of four health education packages
Source: PLoS One. 2023 Sep 8;18(9):e0283405. doi: 10.1371/journal.pone.0283405 (PMC10490887; doi:10.1371/journal.pone.0283405)
Supplement: S1 File — (PDF) [file pone.0283405.s001.pdf]

Lesson plan HE

- **Package 1: Disease Surveillance**

COVID-19

In this package we will discuss the key points of COVID-19, the cause, transmission, prevention methods and treatment. នៅក្នុងកញ្ចប់នេះយើងនឹងពិភាក្សាអំពីចំនុចសំខាន់ៗនៃ COVID-19 ដែលជាមូលហេតុមធ្យោបាយចម្លងវិធីសាស្រ្តបង្ការ និងការព្យាបាល។

Before we start we want to discuss why involvement of VMWs is important for COVID-19

Why is community involvement and also involvement from VMWs important for COVID-19?

-Help detect early outbreaks and new infections

-Reduce practices that increase risk of infection

-Inform the community about COVID-19 and the risks

-Identify and manage false information

COVID-19 is a virus that was first discovered in China and has since spread around the world and become a pandemic.

Transmission

COVID-19 is mainly spread by air droplets that come from the nose or mouth of an infected person when they cough/sneeze/talk/laugh. These droplets carry pieces of the virus and can infect people they come in contact with. Most droplets travel around 1 meter before falling on the ground. This is why social distancing is encouraged. People can also get infected with COVID-19 if they touch surfaces where droplets have landed. COVID-19 can also be transmitted if a person has contact with feces of an infected person. This is why it is important to wash your hands after using the toilet.

When a person becomes infected there will be an incubation time (time until a person develops symptoms). The incubation time can last from 1-14 days, usually it is within 5 days. During the incubation time a person can transmit the virus to other people.

Symptoms

The most common symptoms are fatigue, fever, and coughing. People may also experience a runny/stuffy nose, sore throat, muscle aches, headache or diarrhea/vomiting. Some people are asymptomatic, which means they have no symptoms. Children and adults usually have mild

symptoms. But elderly or people with health problems might get very sick. They can develop chest pain or difficulty breathing. If a person gets very ill or develops chest pain or difficulty breathing it may be a sign that the sickness has progressed and they should visit the healthcare center as soon as possible.

Prevention

There are some preventive measures that can be taken, however they do not guarantee that a person will nog get infected with COVID-19

-Social distancing of 1-1.5 meters

-Wearing masks, especially in crowded places

-Washing hands with clean soap and water or use disinfectant. After using the bathroom, after touching dirty surfaces, after returning home or after having contact with people

-If a person has symptoms similar to COVID-19, get tested and avoid contact with other people

-Disinfect surfaces in the house with alcohol (tables, door handles)

-vaccination is also a possibility. There are currently 5 vaccines: moderna, pfizer, astrazeneca, johnson and sinovec. It is highly recommended that people get the vaccine. The vaccine does not guarantee that someone will not get infected however if a vaccinated person does ge infected the risks of developing severe illness and/or ending up in the hospital are dramatically reduced.So it can protect a person from becoming very ill and getting complications from COVID-19 infection.

How to wash hands?

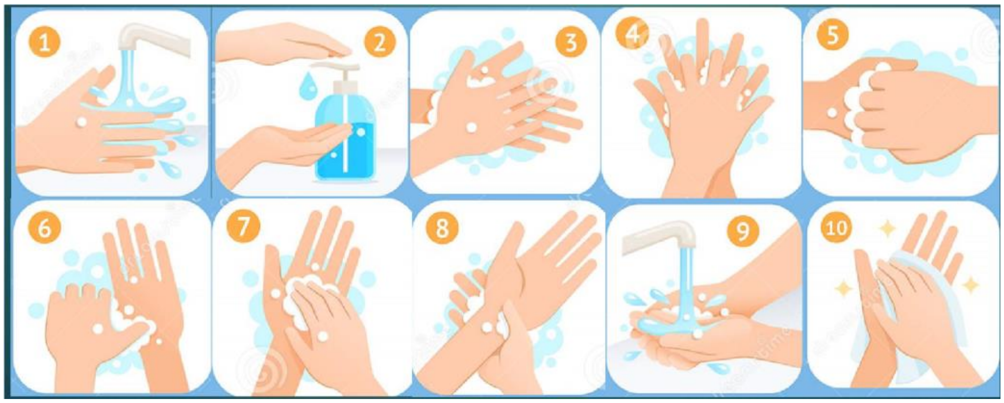

Use soap and clean water and wash hands for at least 40 seconds.

What should a person do if they test positive for COVID-19?

-Self isolation

This means that a person should isolate themselves from all family members and try to stay in a separate room for 14 days. Reduce use of shared spaces, such as the bathroom and kitchen. If they have to use the bathroom and leave the room they should wear a mask and keep a distance of at least 1.5 meters from other family members. Also make sure to wash hands thoroughly and disinfect the bathroom after using it.

During self isolation it is important that the house is well ventilated, so try to keep as many windows open as possible.

- If a person has a runny nose, coughing, headache, muscle aches, fever or a sore throat they should monitor the symptoms at home and check their temperature twice a day
- If they develop a very high fever, difficulty breathing, chest pain or severe diarrhea/vomiting they should contact the healthcare center.

Treatment

Unfortunately there is no specific treatment for COVID-19. The treatment at home can consist of combatting the symptoms for example, using paracetamol to reduce fever or anti-diarrheal drugs to combat diarrhea. Furthermore, to help with nasal congestion patients can use hot water in a bowl to steam. If someone has diarrhea ORS can be given to supplement fluids, it is also good to encourage patients to rest as much as possible and drink plenty of fluids (soda, fruit beverages)

If a person get very ill and has symptoms such as chest pain, altered mental state, difficulty breathing they have to go to the healthcare center. There they can receive supportive care, such as oxygen or i.v. fluids. If the breathing difficulties progress a patient might have to get intubated

Recovery

After a person has had an infection with COVID-19 they can suffer complications. If a person was severely ill and in the hospital they may notice that they have prolonged symptoms of shortness of breath, bad condition, loss of muscles. Some of these symptoms can dissapear with time, however sometimes they do not.

Dengue

During this presentation we will discuss the key points of dengue fever, transmission, prevention, treatment and complications

Dengue fever is a viral infectious disease that is transmitted to humans through the bite of infected mosquitos. There are two main types of tiger mosquitos that can spread dengue.

These mosquitoes lay their eggs in bodies of water and those eggs become mosquitos who can also infect people with dengue. This is why it is very important to not leave bodies of water exposed because mosquitos will lay eggs and the cycle will continue. In Cambodia dengue is prevalent during the rainy season due to more exposed bodies of water where the mosquitos can lay their eggs.

Dengue can affect adults and children and if not treated properly about 1.5% of people can die due to dengue. 26% develop severe dengue fever.

Symptoms

Fever, muscle aches, bone pains, headache, pain behind the eyes, nausea and vomiting. Patients with dengue can also develop petechiae, this is when a person has very small red rashes around their body. When one presses the red dots it does not disappear

Mild dengue fever

Incubation period is around 4-6 days

The fever starts quickly accompanied by a headache. Within 24-48 rashes will appear on the body especially on the face and limbs. The other symptoms mentioned above may also follow; nausea/vomiting, muscle aches.

Severe dengue (hemorrhagic fever)

Some people may develop severe dengue and this condition can be fatal. It is important that if you recognize these symptoms you refer the patient to the healthcare center immediately.

Severe abdominal pain, persistent vomiting, rapid breathing, bleeding gums or nose, fatigue, restlessness, blood in vomit or stool

Prevention

There is no vaccine against dengue. Preventive measures that can be taken to not develop dengue are:

Using bug spray

Wearing long sleeves and pants to not get stung by mosquitos

Sleeping under a net

People should eliminate all bodies of water such as car tires, cans, containers, jars, basins, bottles and other water containers around the home to prevent the mosquitos from laying eggs.

Treatment

There is no specific treatment for dengue; it consists of alleviating the symptoms. At home patients should drink plenty of fluids, in case of dehydration they can ingest ORS, soda, fruit juices. Paracetamol can be used to reduce the fever. NSAIDS such as ibuprofen should not be given !!! as this can have severe consequences for patients who develop severe dengue.

Patients with signs of severe dengue should immediately be transported to the healthcare center for proper treatment. In the hospital they may get i.v. fluids and further treatment.

- **Package 2:**

Expanded programme on Immunization (EPI)
https://immunizationdata.who.int/pages/schedule-by-country/khm.html?DISEASECODE=&TARGETPOP_GENERAL=

During this presentation we will discuss the benefits of vaccination and the vaccines each person must receive.

Benefits of vaccination: getting vaccinated prevents someone from developing a certain disease and developing complications due to the disease. It decreases morbidity but also prevents deaths.

There are a total of 11 vaccines that we are going to discuss. All of these vaccines are implemented in Cambodia from birth.

1. Tuberculosis
2. Hepatitis B
3. Polio
4. Tetanus
5. Pertussis
6. Diphtheria
7. Bacterial meningitis/pneumonia
8. Measles
9. Smallpox
10. Rubella
11. JE encephalitis

1.Tuberculosis

-Tuberculosis is caused by a bacteria

-Transmission occurs through air droplets when an infected person talks/laughs/sneezing or coughs in close proximity of another person

- Symptoms include: fever, weight loss, coughing, chest pain and night sweats. TB can be fatal.

-Getting vaccinated can protect against TB

2. Hepatitis B

- Is an infection caused by a virus
- It can be transmitted due to sexual contact with an infected person or contact with bodily fluids of blood. A pregnant mother can also transmit this disease to her baby

- Patients are fatigued, vomit, have abdominal pain, yellow skin color/eyes. Eventually people develop liver cirrhosis and it can be fatal

-

Getting hepatitis B vaccination can protect against this disease

3.Polio

-Polio is caused by a virus that can cause paralysis and inflammation of the muscles

- Polio is transmitted by ingesting contaminated food or water

-Patients may have a fever, headache and eventually (mild) paralysis and can be fatal

-If a person is vaccinated they are protected from getting polio

4. Tetanus

-Tetanus is caused by a bacteria that can cause muscle spasm

-Tetanus can be transmitted by getting a wound or cut with unclean materials(old and/or rusty) that contain tetanus. Also getting bitten by a dog can give someone tetanus.

-Tetanus has an incubation period of 3-28 days, it causes stiffness of the muscles and seizures and can be fatal

-Tetanus can be prevented by getting the vaccination

5. Pertussis

- It is caused by a bacteria
- Transmission occurs via air droplets when a person talks/cough/laughs or through direct contact
- Symptoms are: persistent coughing, fever, runny nose. In the beginning it could seem like a common cold. In later stages: coughing worsens, vomiting after coughing, fatigue, apnea
- Getting vaccinated can help protect against pertussis

6. Diphtheria

- It is caused by a bacteria that usually affects the tongue/throat/nose
- Transmission occurs via air droplets when a person talks/cough/laughs or through direct contact
- Symptoms are: fever, white coating on the back of the tongue and throat. It can lead to swelling of the glands in the neck. Eventually people can have difficulty breathing and it can cause obstruction of the airway leading to death
- If a person gets vaccinated it prevents them from getting diphteria

-

7. Bacterial meningitis/pneumonia

- Caused by a bacteria called pneumococcen

  It can be spread via air droplets when a person it talking/coughing/sneezing
- Symptoms are coughing, fever, shortness of breath, runny nose, chest pain eventually it can develop into meningitis, which is an infection of the brain this can cause severe symptoms like altered mental state, not reacting and can be fatal
- Getting the vaccine can protect you from this disease

8. Measles

- This is an infection caused by a virus
- Transmission occurs via air droplets when a person talks/coughs/sneezes
- Symptoms are a fever, runny nose and a red rash appears withing 3-5 days of the first symptoms. It starts at the face and head before spreading to the rest of the body, torse etc
- Getting vaccinated can protect against measles

9. Smallpox

-is caused by a virus

- It is transmitted via air droplets air droplets when a person coughs/ talks/ sneezes

-symptoms include fever, headache, vomiting. Three days later a rash will appear first on the face, hands and forearms and afterwards it will spread to the trunk of the body. Lesions may also develop in the mucus membrane of the nose and mouth.

-If a pregnant woman is infected it can effect the baby causing birth defects such as blindness, mental retardation or premature death

-Vaccination can protect against this disease

10. Rubella

- Viral infection
- Transmission occurs via air droplets when a person talks/coughs/sneezes
- Symptoms are fever, nausea, conjunctivitis and a rash that lasts 1-3 days, swollen glands in the necks and behind ears. Pregnant women who get infected can pass the virus to the unborn baby which can be fatal
- Getting vaccinated can prevent against this disease

-

11. Japanese encephalitis

- It is a viral infection
- Transmission occurs via a bite of a mosquito
- Symptoms are high fever, headache, neck stiffness, disorientation, seizure, paralysis and it can be fatal. The people who survive the infection suffer permanent intellectual or neurological problems such as paralysis, recurrent seizures of inability to speak

  Getting the vaccine can prevent people from developing this disease

Discuss with the VMWs that they should encourage community members to visit the nearest healthcare center to get all of their vaccines. NEwborn babies must go to the healthcare center after they are born multiple times to get all of the vaccines. Show them this schedule of when and at how many months children should get vaccinated.

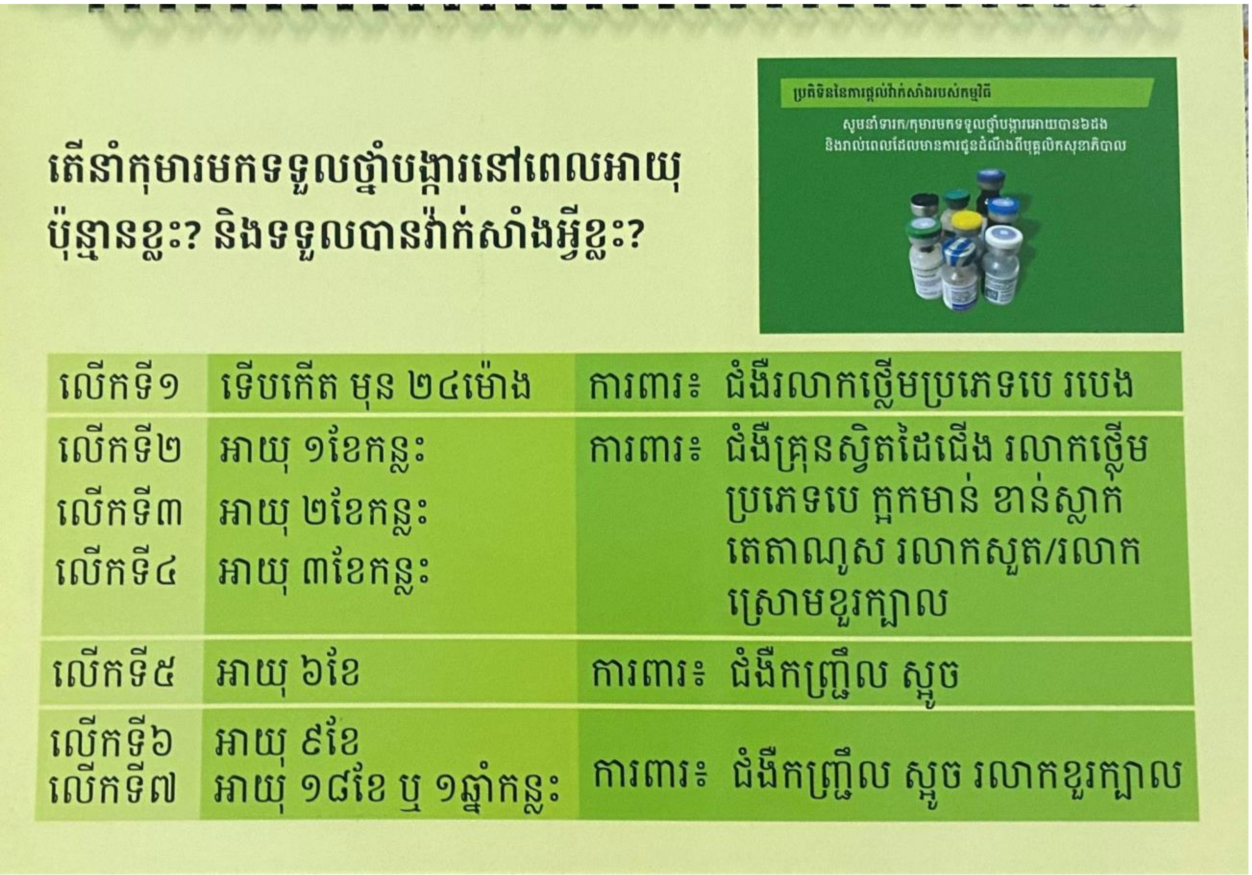

Try to advise VMWs to give education in the village especially to mothers/fathers with young children to encourage them to let the children go to the healthcare center and get their

-

vaccines. Explain the different types of vaccines and the consequences if a child is not vaccinated.

Please immediately report and encourage patients to go to the nearest healthcare center in any of the following cases:

1. High fever and a red rash
2. Constantly coughing difficulty breathing and after coughing, vomiting (at least for 2 weeks)
3. Sore throat with gray mucus membranes sticking to the throat and difficulty breathing

4. Children younger than 15 years where paralysis may occur suddenly
5. If a baby that is younger than 28 days dies
6. Children under the age of 15 years with fever, dizziness, fainting or seizures

Antenatal Care (ANC) https://www.who.int/health-topics/contraception#tab=tab_1

During this presentation we will discuss the importance of antenatal care which is the care women receive during their pregnancy. We will also be discussing how to have safe sex and sexual transmittable diseases.

Sex is something that occurs between a man and a women. It is important to know that having unprotected sex has consequences. Having unprotected sex means having sex without using contraception. Contraception are methods to avoid pregnancy, besides avoiding pregnancy some contraceptives can also protect against sexual transmittable diseases.

Which type of contraceptives are there? Condoms, the pill, IUD, contraceptive ring.

All of the above can prevent pregnancy if used while having sex, this way a person can plan when to have a child and doesn't run the risk of getting pregnant everytime they have sex. If a person does not want a child it is highly recommended they use contraceptive measures.

A condom should also be used if a person is having sex with someone that does not know if they have sexual transmittable diseases. A condom creates a barrier which prevents sexual transmittable disease from spreading to the other person. Examples of sexual transmittable diseases are: Herpes, Chladydia, HIV, syphillis or ghonorrea. HIV and syphillis can be fatal, this is why it is very important to use contraceptives.

When a women gets pregnant they must receive check ups during their pregnancy and also take certain supplements. When a woman thinks she might be pregnant she must take a pregnancy test, preferably more than once to check if she is actually pregnant. If it is positive they must attend the healthcare center to receive: a tetanus shot (if they have not received one in recent years. Furthermore, they must take supplements such as folic acid and iron tables. When a pregnant women is more than 3 months pregnant they take deworming pills.

Women in Malaria prone areas should be advised to sleep under mosquito nets and use mosquito sprays to avoid getting stung by mosquitos.

During the consultation at the healthcare centera woman must also make an appointment for HIV testing

Birth preparation

VMWs should discuss with pregnant women where they plan to give birth and encourage them to go to a healthcare center when they have to give birth. Furthermore, they should also discuss saving money to be able to provide for when they have to give birth and for the post partum project. Every pregnant woman should find a midwife in the area.

The VMWs can explain to a pregnant woman that labor can come at any time and they should think about transportation methods(to the healthcare center) for when they go into labor.

Make sure to discuss the signs of going into labor so that the pregnant women can rexognize this when the time comes, such as: pains that come and go and keep getting stronger, eventually with intervals of 5 minutes. Water breaking or losing a mucus plug.

Pregnancy care

Encourage pregnant women to visit the healthcare centers for their check-ups. To take iron supplements, folic acid and deworming pills. Tell them that they have to eat enough food, avoid strenuous activities and enough rest. Do not use over the counter medicine without consulting with the doctor. Avoid eating food that is raw (such as raw meat) or not properly cooked. MAke sure to wash fruits and vegetables thoroughly.

Danger signs during pregnancy

Discuss with the women if they know the danger signs during pregnancy and when they should get help?

Discuss the danger signs with them:

- High fever
- Heavy vaginal bleeding
- Constant very strong abdominal pain
- Rupture of amniotic sac (water breaking) without any abdominal pain
- Not feeling the baby kick for a long period of taking

Danger signs during labor/ prolonged labor:

- Constant severe abdominal pain
- Seizures
- Severe headache
- Blurred vision
- Swelling of feet, the face and/or arms
- Very heavy bleeding at the beginning of the labor

If any of these signs present themselves the patient must be transported to the healthcare center as soon as possible.

Newborn care

Newborns are very fragile and need help to keep their bodies warm: After the baby is born the baby has to be wiped and put on the mother's chest to receive warmth. It is important to cover the baby with a clean towel. Newborn babies should always wear layers to keep warm. Do not bathe the baby immediately after birth.

The umbilical cord of the baby has to be kept clean and dry until it falls off. The baby should be taken to the healthcare center to receive the proper vaccinations after birth

- **Package 3:**

Hygiene & Sanitation

Slide 1:

During this training session we will discuss the importance of hygiene and sanitation. Furthermore we will give a few examples on how to improve hygiene and the key points of sanitation.

Slide 2:

Hygiene and sanitation are very important for the well being of human beings. Health and hygiene depend on availability of clean water and proper sanitation. Many diseases can be caused due to lack of sanitation and good hygiene. By improving hygiene and sanitation it will reduce the risks of contracting diseases.

Slide 2:

Personal hygiene is when someone takes care of themselves. This can be done by bathing/showering on a daily basis with soap and clean water. Brushing the teeth on a daily basis with toothpase and clean water, in the morning when you wake up and in the evening before you go to sleep. Furthermore, it is important to wear clean clothes everyday and also wear shoes when walking outside on the streets. Other steps that can be taken to improve personal hygiene is by cleaning and trimming the fingernails once they accumulate dirt of get long.

Slide 4:

For personal hygiene it is also very important to maintain cleanliness. Everyday our hands come in contact with various objects and therefore various bacteria throughout the day. To minimize the chances of contracting disease and maintain proper hygiene it is important to wash hands often during the day. For example, after returning home, after using the toilet, before and after cooking or after you have touched something that might not be clean.

To properly wash hands you will need soap and clean(!) water. As you can see in the instructions, first you will make your hands wet with water then followed by soap, afterwards you should wash your hands for 40-60 seconds. Wash the soap off with water and dry hands with a clean piece of fabric.

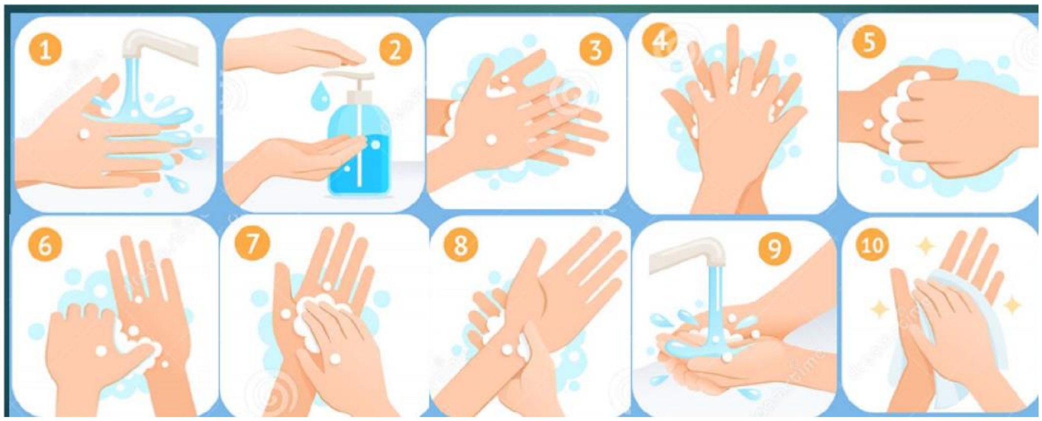

Slide 5

During this presentation we have mentioned the use of clean water. But can one ensure that the water is clean? Use water that is clear, do not use water that has already been used for something else as this water might be dirty. Dispose of dirty water after it has been used.

For example, if you have used water to wash the dishes or clothes, this water has to be disposed afterwards and not be repurposed.

Slide 6:

It is also important to use (clean) water to rinse vegetables/fruits before cooking them. If tap water is available it should always be boiled before using it to cook or drink. The reason why it is to important to have (clean) water and to use it in this way is because unclean water may be contaminated, which can cause illnesses such as:

-Diarrhea(due to E. Coli(bacteria), Norovirus or parasites)

-Hepatitis A

-Respiratory disease

-Thypoid Fever

Slide 7:

By contracting a virus, bacteria or parasite someone can get very ill and develop symptoms some of these organisms lead to diarrhea. Which can make someone ill and lead to dehydration.

Hepatitis A is a virus that can be contracted when ingesting contaminated food/water. The symptoms include: fever, dark-colored urine, abdominal pain, nausea and yellow eyes/skin tone(jaundice)

Typhoid Fever is a bacteria that gets contracted by ingestion of contaminated food/water. The symptoms include fever, diarrhea, fatigue, muscle pain and nausea.

Respiratory disease can be contracted by a virus or a bacteria, symptoms include fever, coughing, chest pain and nasal congestion

Slide 8

If some community members can not boil water in their house, advise them to look for other common water sources. Some examples may be collecting water at a well. The water collected from a well can safely be ingested and used for other purposes such as bathing and cooking.

Slide 9

Community members should be advised to not swim or digest water from rivers. Although the river might have clear water and look clean, it can still contain bacteria/parasites. Therefore ingesting water from a river can increase the risk of developing illnesses. To try and keep the river as clean as possible it is advised to not dispose of any garbage in or near the river as this can pollute it and eventually cause clogging.

Slide 10

It is also important to not throw garbage at undesignated areas such as : on the streets or in the backyard. Encourage community members to look for proper garbage disposal areas such as bins/garbage cans. It is also important to dispose of garbage in the house since this may lead to an unsanitary environment. If garbage is not disposed of it will attract flies and other insects/animals. This can eventually lead to unsanitary conditions and infestation of rats. This can then cause even more disease. If someone is bitten by a rat they can get very sick.

Slide 10

During this presentation we have mentioned the importance of properly washing fruits and vegetables. Furthermore it is important that food around the house is properly covered. COmmunity members can do this by using a lid or store food in a pot/fridge or tupperware. It is important that (raw) meat be stored in cool places such as a fridge to avoid insects from getting on it. If (raw) meat is unrefrigerated for a long time >2 hours it can become spoiled and unsafe to eat. If flies or other insects get on the meat they might also contaminate it. Before eating meat it is important to properly refrigerate it and cook it with high temperature to get rid of any bacteria. **Slide 11**

Community members should be advised to strictly use the toilet for urination/defecation. Use water for flush the toilet after using it. Do not urinate or defecate in public areas as tis will attract flies and other animals and will lead to unsanitary conditions.

- **Package 4: Disease Management**

Typhoid Fever https://www.who.int/news-room/fact-sheets/detail/typhoid

Slide 1: introduction

During this lesson we will give a brief explanation of the key points of Typhoid fever. We are going to discuss transmission, cause, prevention methods and treatments for Typhoid fever

Slide 2: Cause and transmission

Tyhpoid fever is a disease that anyone can get infected with. It is caused by the bacteria Salmonella Tyhpi which can be present in contaminated water and foods. People get infected by ingesting contaminated water and/or food.

Slide 3: Symptoms

When a person gets infected they start to feel ill and develop certain symptoms. Most people develop fever and may also feel fatigued. Other common symptoms are nausea, vomiting and abdminal pain. People may also develop diarrhea or constipation. Some people also develop a rash, which can be seen on the abdominal area. Patients can get very ill and this disease can be fatal.

Slide 4: Prevention

There are certain measures that can be taken to prevent contracting Tyhpoid fever, such as:

Cook food properly, make sure that meat is not raw and food is heated up wel

Wash vegetables and fruits thoroughly with clean water before eating them

Wash hands with soap and clean water before cooking food, eating food or after using the toilet

In some cases vaccination can be used to protect against Typhoid fever, however it does not prevent 100% from contracting the disease

Drink water that is clean from a sanitary source , for example from a well and not from a river

Slide 5: Treatment

The only treatment that can cure Typhoid fever are antibiotics. In the meantime paracetamol can be give to reduce fever. ORS can be given to combat dehydration. Anti- emetics or antidhiarreal drugs can also be used to relieve symptoms.

Slide 6:

When someone is suspected to have this disease it is important to refer them to the healthcare center to get diagnosed and get proper treatment. Without proper treatment Typhoid Fever can be fatal!

If someone has a fever that does not go down, has an impaired mental state and is constantly vomiting/dhiarrea. Advise this person to visit the healthcare center as soon as possible to avoid dehydration and progression of the disease.

First aid

Slide 1: Introduction

During this presentation we will briefly discuss a few key points of first aid. We will be discussing bleeding and how to stop it, wound care and burn injury's.

Slide 2: Bleeding

Often people get in an accident of cut themselves with sharp objects, in these cases bleeding can occur. It is important to be able to stop the bleeding as soo as possible. If it is a small wound this can be done by applying pressure on the wound untill the bleeding stops.

Here we will give an example on how to stop the bleeding

Slide 3: Bleeding

Sometimes it can be a big cut and the bleeding will be severe. In these cases it is important to apply pressure until the person has reached a healthcare facility. If the presure is not properly applied it can be fatal. You can apply pressue by using your hands ot a piece of fabric. In some cases an object such as a belt can be used to stop the blood flow.

Here we will give examples on how to stop a bleeding with hands and with a belt.

Slide 4:Wound care

When a person has an open wound due to a cut or an accident it is important to disinfect the wound. This can be done with antiseptic solutions or water. The second step if to cover the wound with a band-aid (if it is a small wound) to prevent any dirt from getting on it. If it is a big deep wound the person should go to the healthcare center as stitches may be necessary.

Slide 5: Wound care

In some cases a wound can get infected and not hear properly. Signs of an infected wound are:
-Redness around the wound

-Pain

-Pus or cloudy fluid draining from the wound

-Swelling around the wound

-Fever

Slide 6: Wound care

If someone has signs of an infected wound they must go to the healthcare center for proper treatment. During this slide we will also show some examples of what an infected wound may look like.

Slide 7: Burn

A burn is an injury to the skin caused by heat, electricity or chemicals and it requires proper treatment.

Slide 8: Burn

Burns can be classified in three stages

-First degree burn: this burn is superficial and only affects the skin(First picture)

-Second degree burn: this burn causes damage beyond the skin and causes blistering (second picture)

-Third degree burn- this burn destroys multiple layers of the skin (third picture)

Slide 9: Burn

Third degree burns can be fatal if not treated in time. Due to destruction of the skin it can cause dehydration and eventually shock. A person should immediately go to the nearest healthcare center if there is suspicion of a third degree burn.

Slide 10: Burn

The first stop to treat a burn injury is to cool the burn with cool/lukewarm water for 10-20 minutes. It is important to use ice or cold water. A burn should be covered with non-adherent material. Paracetamol can be administered to alleviate the pain.

Slide 11:Burn

A patient should be transferred to a healthcare center immediatly if:

- The burns are larger then the size of the persons hand

- If the burns are on the neck, face, feet and genitals
- If the burns were caused by chemicals or electricity
- If the patient is feeling ill
- If the burn wound has signs of infection
- If the patient is a child
- If a blister is bigger than 10 cm - If it is a third degree burn

Case : Bleeding

Case : Wound care

Case : Burn injury

Respiratory Infection

Slide 1:

Slide 2:

Respiratory infection refers to an infectious disease involving the respiratory tract. It can be divided into two categories: upper and lower respiratory tract infection

Slide3:

On this slide we can see the upper respiratory tract and the lower respiratory tract

Slide 4:

A respiratory infection can be caused by a virus of bacteria and transmission can occur via air droplets, direct physical contact or very close contact. For example, at kindergarten, daycare, schools, tours or public transportation

Slide 5:

When a person gets infected they develop symptoms such as: Coughing, sneezing, sore throat, headaches, muscle aches, stuff/runny nose, fever. Some more severe symptoms include shortness of breath, chest pain or wheezing which indicate that a person is having difficulty breathing which can lead to fatality

Slide 6:

Symptoms susch as high fever hortness of breath, chest pain or wheezing which indicate that a person is having difficulty breathing which can lead to fatality. These symptoms may be an

indication that the infection has progressed and a person could have developed pneumonia or bronchitis.

Slide 7:

It can be difficult to recognize shortness of breath in children. A few symptoms can be nasal flaring, wheezing, interrupted laughing/crying or exaggerated abdomen breathing

Slide 8:

Pneumonia and bronchitis are both infections of the lungs. They indicate progression of the disease and that the disease has spread out through the respiratory system. Patients who have this disease can get very ill and it can be fatal if not treated properly.

Slide 9:

There are a few ways to treat these symptoms at home, such as: Drinking a lot of fluids to prevent dehydration, and paracetamol to reduce the fever. Steaming with hot water for 5-10 minutes at a time can help with nasal congestion.

Slide 10:

If a person has symptoms of severe illness or prolonged symptoms they should visit the healthcare center for further treatment/diagnostics. A person may receive oxygen or antibiotics to combat the disease

Slide 11:

Dehydration

Slide 1:

During this training session we will discuss the key points dehydration

Slide 2:

Dehydration is a condition caused by excessive loss of fluids. When a person is dehydrated the loss of fluids is greater than fluids being taken into the body. Often dehydration is secondary to an illness or disease

Slide 3:

Dehydration can have several causes such as:

-Vomiting

-Diabetes

-Heatstroke

-High fever >38,5 (due to excessive sweating)

-Medication(diuretics, laxatives)

Slide 4:

Symptoms of dehydration may include:

Dry mouth, reduced urine, dark colored, strong smelling urine, increased thirst, dizziness, headache, loss of weight, exhaustion

Slide 5:

Symptoms in infants can be very different than symptoms in adult. Some important symptoms of dehydration in children are: crying without tears, less wet diapers a day, deep rapid breathing, sunken eyes, dry and wrinkled skin.

Explanation on how to recognize these symptoms with pictures

In case you notice these symptoms in a child the child should be brought to the healthcare center immidiately

Slide 6:

Dehydration can be very dangerous and patients may get severely ill. PAtients may get severely dehydrated, have diarrhea or vomiting longer than 2 weeks, or diarrhea more than 6 times a day, consistently vomiting, or an altered mental state.

Slide 7:

Oral redhydration solution (ORS) is important when treating patients with dehydration. ORS is a mixture of important substances that the body needs which have been depleted due to diarrhea.

How to mix ORS: pour all of the powder from one packet into a jar/bottle, measure the amount of litres of clean water. Pour the water into the bottle/jar untill all the powder has dissolved.

Slide 8:

What should someone do when someone has mild dehydration?

Rest, stay out of the sun, drink plenty of fluids, not only water but also things with electrolytes such as ORS, chicken bouillon, fruit, juices or soda. Anti emetics/ diarrheall drugs can be administered to try and stop diarrhea or vomiting

Slide 9:

Severe dehydration→ ORS, patients should go to the healthcare center immediately to receive fluids via i.v. and other treatment
