# Supplementary material for: Expanding the role of village malaria workers in Cambodia: Implementation and evaluation of four health education packages
Source: PLoS One. 2023 Sep 8;18(9):e0283405. doi: 10.1371/journal.pone.0283405 (PMC10490887; doi:10.1371/journal.pone.0283405)
Supplement: S2 File — (PDF) [file pone.0283405.s002.pdf]

Standard interview guidelines for VMWs and healthcare workers

Project name: Expanding the roles of village malaria workers: Operational research in Cambodia

Note: *Use of this guide is expected to be flexible and will be adapted for FGDs and SSIs. The guide is expected to help interviewer stay within the confines of the themes guided by the research question. Questions included below are only examples and should be adapted during the discussions/interviews. Probes and potential questions under the themes can be re-phrased and asked in ways you as an interviewer think appropriate. You may add questions/probes to collect additional and important information*

1. Introduction and background questions

Thank you very much for participating in this interview. To begin with, could you tell me something about your background and current work?

**General Information**		
**Socio-demographic characteristics of respondents**	1. Age	
	2. Gender	Male ☐ Female ☐ Other ☐
	3. Occupation	
	4. Workplace	e.g., xx health centre.
	5. Village Name	
	6. Qualification/Education	
	7. Categories of respondent	1. Health officials/policymakers ☐<br>2. VMWs ☐<br>3. Community members ☐<br>4. Others ☐ [Specify______________________

2. Health sector gaps

As part of this project, we would like to gain a better understanding of challenges to health care delivery in remote rural communities. What are your views?

<u>Probes</u>:

- Focus on the management of febrile illness, including diagnosis, referral, and treatment (with examples)

- Consider barriers in access to services across different dimensions: geographic accessibility, availability, affordability, and acceptability

- Encourage discussion of challenges associated with specific population groups (e.g., ethnic minorities, women) and health concerns

- Why do you think there are gaps around management of febrile illnesses? What are potential solutions? Have the solutions you outlined been tried? Why?

3. Expanding the role of village health workers or your (VMWs) roles

Let's think about the challenges in management of febrile illnesses in rural communities. Do you think you or village health workers could be utilised to address these challenges? If so, how, in what capacities?

Probes:

- What are the current roles and responsibilities undertaken by village health workers? Do you think village health workers are able to fulfil their current roles and responsibilities?
- Do you ever think or have you heard your colleagues or VMWs feeling the current roles and responsibilities burdensome?
- Encourage retrospective analysis of previous experiences with village health workers, particularly for the management of malaria
- Encourage a reflection on specific challenges to, and opportunities for, the expansion of VHWs, including policy and operational challenges
- Mention specific issues such as financing and sustainability, integration in the wider health sector, and motivation
- How would you propose strategies/ways to sustain the expansion of roles and responsibilities to VMWs? (e.g., training, supervision, incentives-both monetary and non-monetary, extent of expanding the roles and responsibilities). Please share your practical ideas.

4. Community-based interventions to support other health activities

The last point we would like to discuss is the potential use of village malaria workers to support other health activities in remote communities. Do you think it would be useful?

Probes:

Encourage discussion of the following points:

- Let's discuss how we can further expand the roles of VMWs beyond febrile illnesses. Do you think VMWs can be trained and provided responsibilities on 1) management of community hygiene and sanitation; 2) disease surveillance; 3) EPI and ANC support and 4) management of mild common illnesses (these could be based on the respondents' perceptions. In case you

would like to clarify, you could give examples of common cold, viral infections, and self-limiting illnesses). Probe them one-by-one.

- How can we utilise VMWs to deliver key messages in the communities related to general hygiene and sanitation? Encourage to share examples.

- Do you think VMWs can conduct disease surveillance? Have they done before (during emergencies)? How do you anticipate their expanded roles in conducting disease surveillance (what do you see challenges and opportunities)?

- How programs related to the current topic conducted in the community ? Who does it? Do you think VMWs can support these programmes? How can they support? To what extend can they support?

- How are mild common illnesses dealt in communities? Who is taking care of these illnesses currently? Do you think VMWs can undertake the management of common illnesses? To what extent can they support the management of common illnesses?

- Can VMWs be trained to identify suspected cases based on symptoms, temperature measurement and, when available, RDTs?

- Do you think VMWs can be trained to suspect various diseases (beyond malaria and febrile illnesses) and refer confirmed cases to the health facilities?

- What other additional tasks do you think VMWs can perform at the community level?

- Can you reflect on what could be the potential barriers and facilitators when expanding their roles?
- How do community members react to the advice/education of VMWs? Do they follow the advice? Do they listen? Do they think it is important?

- Do community members willingly go to VMWs or seek help elsewhere? For example, with traditional medicine, self medication etc?

5. Health education training sessions

Probes:

- How did you experience the health education training session?

- What is your opinion on the topic of the health education training session ?

- What is your opinion on the information provided throughout the health education training session? Do you think it is relevant? Do you find it interesting? Is it suitable for VMWs or community members?

- Do you have any suggestions how the training sessions can be improved? It could be based on on the information provided , the setting, the materials

- Do you think you can implement this new information in your work as a VMW? If so, how will you implement it in your work as a VMW ?

- Which additional activities /tasks will VMWs be able to perform, besides tasks that they are currently doing?

- Which additional activities/tasks do you think VMWs would like to learn more about and would be useful?

- Have you received education on this subject before?

- What are other topics you would like to receive education on?

6. Conclusions

Thank you very much. Would you have anything to add? Do you have any questions? Would you have any suggestions about specific areas of investigation we should consider in future interviews? Could you name other informants we should talk to?
