# Supplementary material for: Expanding the role of village malaria workers in Cambodia: Implementation and evaluation of four health education packages
Source: PLoS One. 2023 Sep 8;18(9):e0283405. doi: 10.1371/journal.pone.0283405 (PMC10490887; doi:10.1371/journal.pone.0283405)
Supplement: S3 File — (PDF) [file pone.0283405.s003.pdf]

Standard interview guidelines for community members Health education and novel RDTs for VMWs
Project name: Expanding the roles of village malaria workers: Operational research in Cambodia.

Note: *Use of this guide is expected to be flexible and will be adapted for FGDs. The guide is expected to help interviewer stay within the confines of the themes guided by the research question. Questions included below are only examples and should be adapted during the discussions/interviews. Probes and potential questions under the themes can be re-phrased and asked in ways you as an interviewer think appropriate. You may add questions/probes to collect additional and important information.*

Introduction and background questions

Thank you very much for participating in this interview. To begin with, could you tell me something about your background and current work?

**General Information**		
**Socio-demographic characteristics of respondents**	1. Age	
	2. Gender	Male ☐Female ☐ Other ☐
	3. Occupation	
	4. Workplace	
	5. Village Name	
	6. Education completed	
	7. Categories of respondent	Health officials/policymakers ☐<br>VMWs ☐<br>Community members ☐<br>Others ☐ [Specify____]

We will first start with questions regarding the health education packages, including questions about their relevance, benefits and challenges. Then we will ask you questions regarding the rapid diagnostic tests provided to the VMWs.

Relevance of VMWs

As part of the expansion of the VMW roles, they have received health education sessions on disease management, disease surveillance, hygiene and sanitation and/or EPI & ANC.

2A. How important are these topics for your communities?

<u>Probes:</u>
Why is it important to educate community members on these four topics?
Are there other health issues, that are not covered by the health education packages, relevant to the community?
Any health challenges you see in the community that is not being addressed in one of our four topics (Disease Management, Disease Surveillance, Hygiene & Sanitation or EPI & ANC)?

2B. Do you think VMWs are contributing to addressing these challenges?

<u>Probes:</u>
Are the VMWs the best way to educate the community members on the topics (disease management, disease surveillance, hygiene and sanitation and EPI & ANC) that have been chosen?
Do you see any other potential people who could fulfil the roles they have been given?

Providing information to the community members

One of the additional tasks implemented since starting the healthcare education packages is the ability to relay the information to community members. We would like to evaluate this process.

Do you feel that the VMWs are capable of providing information to the community members?

<u>Probes</u>
Have VMWs started activities regarding one (or more) of the topics in your community?
For example: explained certain topics during a village meeting or spread flyers/posters throughout the community.
Are there ways we can improve educating the community members through the VMWs?

Benefits and Challenges

We would like to ask your opinion on the benefits and challenges of having VMWs follow the health education program.

4A. What are, in your eyes, the benefits for the community members?

<u>Probes:</u>
What do you think are the benefits of these education packages for your community?
Where and how has it helped the community to fight off the diseases?
Have you noticed any changes within the community after the VMWs received the information on the topics?

4B. What are some challenges of the VMWs participating in the healthcare education packages?

<u>Probes:</u>
Encourage a reflection on specific challenges for the current expansion of VMWs.
Do you feel like the current roles and responsibilities are burdensome for VMWs?
If so, how or why?

Rapid diagnostic tests

Previously, VMWs only tested for malaria infections in fever patients. Now, to expand their roles, they are also able to test for dengue and CRP (which could indicate a bacterial infection).

5A. What is your opinion of the ability to test for diseases other than malaria?

<u>Probes:</u>

Focus on the ability to test for dengue.
Focus on the ability to test for bacterial infections (through CRP).
Consider the possibility of testing for other diseases in the future.

5B. Would you go to a VMW if you have a fever?

Probes:
Explore if community members prefer consulting a private practitioner or a health centre directly instead of the VMW.
Consider barriers across different dimensions: geographic accessibility, availability, affordability, and acceptability.
Explore if patients are satisfied after a VMW consultation.

Conclusion

Thank you very much. Would you have anything do add? Do you have any questions?
