# Supplementary material for: Expanding the role of village malaria workers in Cambodia: Implementation and evaluation of four health education packages
Source: PLoS One. 2023 Sep 8;18(9):e0283405. doi: 10.1371/journal.pone.0283405 (PMC10490887; doi:10.1371/journal.pone.0283405)
Supplement: S4 File — (PDF) [file pone.0283405.s004.pdf]

Standard interview guidelines for VMWs - Healthcare Education Package

Disease Management

Project name: Expanding the roles of village malaria workers: Operational research in Cambodia.

Note: *Use of this guide is expected to be flexible and will be adapted for FGDs. The guide is expected to help interviewer stay within the confines of the themes guided by the research question. Questions included below are only examples and should be adapted during the discussions/interviews. Probes and potential questions under the themes can be re-phrased and asked in ways you as an interviewer think appropriate. You may add questions/probes to collect additional and important information.*

1. Introduction and background questions

Thank you very much for participating in this interview. To begin with, could you tell me something about your background and current work?

**General Information**		
**Socio-demographic characteristics of respondents**	1. Age	
	2. Gender	Male ☐ Female ☐ Other ☐
	3. Occupation	
	4. Workplace	
	5. Village Name	
	6. Education completed	
	7. Categories of respondent	VMWs
	8. Number of years being a VMW	

2. Relevance

As part of the expansion of your roles as VMWs, you have received health education sessions on Disease Management.

How important is Disease Management for your communities?

Probes:

- Focus on the need to educate community members on Disease Management.
  - Is it relevant to the community, and why?

Do you think you are contributing to addressing these challenges?

Probes:
- Do you think VMWs are able to fulfil their current roles and responsibilities?
- Are the VMWs the best way to educate the community members on Disease Management?

3. Quality of the Education

You have received education on Disease Management.

What do you think of the quality of the education provided during the sessions on Disease Management?

Probes:
- Was the quality of the education as you had expected?
- What did you learn during the session(s)?
    - Did you miss any knowledge or skills?
- Is there something you would have expected to learn but was not included in the session?

4. Providing information to the community members

One of the additional tasks implemented since starting the healthcare education packages is the ability to relay the information to community members. We would like to evaluate this process.

Do you feel capable of providing information on Disease Management to the community members?

Probes:
- Do you use any information/education materials (such as posters/images) to support the delivery the information about Disease Management?
    - Do you use social media to support you?
- Are there ways we can improve educating the community members through you as VMW?

Do you feel like the community members understand the information you provide to them?

Probes:
- Do you feel like people in the community are interested?
- Do you feel like all community members have equal access to the information provided by the education packages?

5. Benefits

We would like to ask your opinion on the benefits of following this education program Disease Management.

What are the benefits of following the education package for Disease Management?

Probes:
- What are for you the benefits of having followed the education on Disease Management?
    - What are or have been motivating factors for you with the expansion of your role?

- Reflect on the challenges (such as, patients not knowing what to do when they are sick) before and after having received the education.
    - Have you noticed any changes after receiving the information?
- What are, in your eyes, the benefits for the community members?

6. Drawbacks

We would like to ask your opinion on the drawbacks of following this education program Disease Management.

What are some drawbacks of using the healthcare education packages?

<u>Probes:</u>

- Encourage a reflection on specific challenges for the current expansion of VMWs
    - Mention specific issues such as transportation, availability of tools to educate community members or motivation (of patient and VMWs themselves).
- Do you ever feel like the current roles and responsibilities are burdensome?

Do you sometimes feel limited in your new role?

<u>Probes:</u>

- Are you missing support to help the community members with the education you have received?
- Do patients want tests or treatment from you for illnesses?
- Any health challenges you see in the community that is not being addressed in one of our four topics (Disease Management, Disease Surveillance, Hygiene & Sanitation or EPI & ANC)?

7. Conclusion

Thank you very much. Would you have anything do add? Do you have any questions?

- COVID-19 less relevant now that the cases are less and less?
- Should COVID-19 be changed for another disease?

Hygiene and sanitation

- Washing / disinfecting hands less relevant? As nationally (and internationally) the past two years there has been a lot of attention for teaching people to wash their hands correctly.

Standard interview guidelines for VMWs - Healthcare Education Packages

Disease Surveillance

Project name: Expanding the roles of village malaria workers: Operational research in Cambodia.

Note: *Use of this guide is expected to be flexible and will be adapted for FGDs. The guide is expected to help interviewer stay within the confines of the themes guided by the research question. Questions included below are only examples and should be adapted during the discussions/interviews. Probes and potential questions under the themes can be re-phrased and asked in ways you as an interviewer think appropriate. You may add questions/probes to collect additional and important information.*

1. Introduction and background questions

Thank you very much for participating in this interview. To begin with, could you tell me something about your background and current work?

**General Information**		
**Socio-demographic characteristics of respondents**	1. Age	
	2. Gender	Male ☐ Female ☐ Other ☐
	3. Occupation	
	4. Workplace	
	5. Village Name	
	6. Education completed	
	7. Categories of respondent	VMWs
	8. Number of years being a VMW	

2. Relevance

As part of the expansion of your roles as VMWs, you have received health education sessions on Disease Surveillance.

How important is Disease Surveillance for your communities?

Probes:

- Focus on the need to educate community members on Disease Surveillance.
    - Is it relevant to the community, and why?
- COVID-19 less relevant now that the cases are less and less?
    - Should COVID-19 be changed for another disease within this education package?

Do you think you are contributing to addressing these challenges?

Probes:

- Do you think VMWs are able to fulfil their current roles and responsibilities?
- Are the VMWs the best way to educate the community members on Disease Surveillance?

3. Quality of the Education

You have received education on Disease Surveillance.

What do you think of the quality of the education provided?

Probes:

- Was the quality of the education as you had expected?
- What did you learn during the session(s)?
  - Did you miss any knowledge or skills?
- Is there something you would have expected to learn but was not included in the session?

4. Providing information to the community members

One of the additional tasks implemented since starting the healthcare education packages is the ability to relay the information to community members. We would like to evaluate this process.

Do you feel capable of providing information to the community members?

Probes:

- Do you use any information/education materials (such as posters/images) to support the delivery the information about Disease Surveillance?
  - Do you use social media to support you?
- Are there ways we can improve educating the community members through you as VMW?

Do you feel like the community members understand the information you provide to them?

Probes:

- Do you feel like people in the community are interested?
- Do you feel like all community members have equal access to the information provided by the education packages?

5. Benefits

We would like to ask your opinion on the benefits of following this education program Disease Surveillance.

What are the benefits of following the education package on Disease Surveillance?

Probes:

- What are for you the benefits of having followed the education on Disease Surveillance?
  - What are or have been motivating factors for you with the expansion of your role (to include Disease Surveillance)?

- Reflect on the challenges (such as, patients spreading a disease to others without knowing) before and after having received the education.
    - Have you noticed any changes after receiving the information?
- What are, in your eyes, the benefits for the community members?

6. Drawbacks

We would like to ask your opinion on the drawbacks of following this education program Disease Surveillance.

What are some drawbacks of using the healthcare education packages?

<u>Probes:</u>

- Encourage a reflection on specific challenges for the current expansion of VMWs
    - Mention specific issues such as transportation, availability of tools to educate community members or motivation (of patient and VMWs themselves).
- Do you ever feel like the current roles and responsibilities are burdensome? (too much work)

Do you sometimes feel limited in your new role?

<u>Probes:</u>

- Are you missing support to help the community members with the education you have received?
- Any health challenges you see in the community that is not being addressed in one of our four topics (Disease Management, Disease Surveillance, Hygiene & Sanitation or EPI & ANC)?

7. Conclusion

Thank you very much. Would you have anything do add? Do you have any questions?

Standard interview guidelines for VMWs - Healthcare Education Packages

Hygiene & Sanitation

Project name: Expanding the roles of village malaria workers: Operational research in Cambodia.

Note: *Use of this guide is expected to be flexible and will be adapted for FGDs. The guide is expected to help interviewer stay within the confines of the themes guided by the research question. Questions included below are only examples and should be adapted during the discussions/interviews. Probes and potential questions under the themes can be re-phrased and asked in ways you as an interviewer think appropriate. You may add questions/probes to collect additional and important information.*

1. Introduction and background questions

Thank you very much for participating in this interview. To begin with, could you tell me something about your background and current work?

**General Information**		
**Socio-demographic characteristics of respondents**	1. Age	
	2. Gender	Male ☐ Female ☐ Other ☐
	3. Occupation	
	4. Workplace	
	5. Village Name	
	6. Education completed	
	7. Categories of respondent	VMWs
	8. Number of years being a VMW	

2. Relevance

As part of the expansion of your roles as VMWs, you have received health education sessions on Hygiene & Sanitation.

How important is Hygiene & Sanitation for your communities?

<u>Probes:</u>

- Focus on the need to educate community members on Hygiene & Sanitation.
    - Is it relevant to the community, and why?
- The past two years there has been a lot of attention for teaching people to wash and disinfect their hands correctly. Do you feel like it is still relevant to inform people on this subject?

Do you think you are contributing to addressing these challenges?

Probes:

- Do you think VMWs are able to fulfil their current roles and responsibilities?
- Are the VMWs the best way to educate the community members on Hygiene & Sanitation?

3. Quality of the Education

You have received education on Hygiene & Sanitation.

What do you think of the quality of the education provided?

Probes:

- Was the quality of the education as you had expected?
- What did you learn during the session(s)?
    - Did you miss any knowledge or skills?
- Is there something you would have expected to learn but was not included in the session?

4. Providing information to the community members

One of the additional tasks implemented since starting the healthcare education packages is the ability to relay the information to community members. We would like to evaluate this process.

Do you feel capable of providing information on Hygiene & Sanitation to the community members?

Probes:

- Do you use any information/education materials (such as posters/images) to support the delivery the information about Hygiene & Sanitation?
    - Do you use social media to support you?
- Are there ways we can improve educating the community members through you as VMW?

Do you feel like the community members understand the information you provide to them?

Probes:

- Do you feel like people in the community are interested?
- Do you feel like all community members have equal access to the information provided by the education packages?

5. Benefits

We would like to ask your opinion on the benefits of following this education program Hygiene & Sanitation.

What are the benefits of following the education packages?

Probes:

- What are for you the benefits of having followed the education on Hygiene & Sanitation?
    - What are or have been motivating factors for you with the expansion of your role?

- Reflect on the challenges (such as, being sick due to bad hygiene and sanitation) before and after having received the education.
    - Have you noticed any changes after receiving the information?
- What are, in your eyes, the benefits for the community members?

6. Drawbacks

We would like to ask your opinion on the drawbacks of following this education program Hygiene & Sanitation.

What are some drawbacks of using the healthcare education packages?

Probes:

- Encourage a reflection on specific challenges for the current expansion of VMWs
    - Mention specific issues such as transportation, availability of tools to educate community members or motivation (of patient and VMWs themselves).
- Do you ever feel like the current roles and responsibilities are burdensome?

Do you sometimes feel limited in your new role?

Probes:

- Are you missing support to help the community members with the education you have received?
- Any health challenges you see in the community that is not being addressed in one of our four topics (Disease Management, Disease Surveillance, Hygiene & Sanitation or EPI & ANC)?

7. Conclusion

Thank you very much. Would you have anything do add? Do you have any questions?

Standard interview guidelines for VMWs - Healthcare Education Packages

Expanded programme on Immunization and Antenatal Care

Project name: Expanding the roles of village malaria workers: Operational research in Cambodia.

Note: *Use of this guide is expected to be flexible and will be adapted for FGDs. The guide is expected to help interviewer stay within the confines of the themes guided by the research question. Questions included below are only examples and should be adapted during the discussions/interviews. Probes and potential questions under the themes can be re-phrased and asked in ways you as an interviewer think appropriate. You may add questions/probes to collect additional and important information.*

1. Introduction and background questions

Thank you very much for participating in this interview. To begin with, could you tell me something about your background and current work?

**General Information**		
**Socio-demographic characteristics of respondents**	9. Age	
	10. Gender	Male ☐ Female ☐ Other ☐
	11. Occupation	
	12. Workplace	
	13. Village Name	
	14. Education completed	
	15. Categories of respondent	VMWs
	16. Number of years being a VMW	

2. Relevance

As part of the expansion of your roles as VMWs, you have received health education sessions on EPI & ANC.

2A. How important is EPI & ANC for your communities?

Probes:

- Focus on the need to educate community members on EPI & ANC.
    - Is it relevant to the community, and why?
- How is particular antenatal care relevant? (probe for safe sex and pregnancy care)
- Encourage discussion of challenges associated with specific population groups (e.g., ethnic minorities, women) and health concerns

2B. Do you think you are contributing to addressing these challenges?

Probes:

- Do you think VMWs are able to fulfil their current roles and responsibilities?
    - Do you see any need for an additional help?
- How are EPI & ANC support programmes in the community normally conducted?
    - Who does it?
    - Do you think VMWs can support these programmes?

3. Quality of the Education

You have received education on EPI & ANC.

3A. We would like you to brainstorm on the health education session.

Probes:

- What is your general impression about the session?
- Discuss about the details of the topics covered (eg. immunization, safe sex, birth preparation) within the health education session.

3B. What do you think of the quality of the education provided?

Probes:

- Was the quality of the education as you had expected?
    - If you thought the quality of education was good or as expected, would you share your opinion on what made it good quality?
- What did you learn during the session(s)?
    - Did you miss any knowledge or skills?
- Based on your experience, are there any better ways to improve the quality of the education?

4. Providing information to the community members

One of the additional tasks implemented since starting the healthcare education packages is the ability to relay the information to community members. We would like to evaluate this process.

4A. Do you feel capable of providing information on EPI & ANC to the community members?

Probes:

- What makes you feel capable of providing information on the EPI & ANC to your community members?
- Do you use any information/education materials (such as posters/images) to support the delivery the information about EPI & ANC?
  - o Do you use social media to support you?
- Are there ways we can improve educating the community members through you as VMW?

4B. Do you feel like the community members understand the information you provide to them?

Probes:

- Do you feel like people in the community are interested?
- What do you think community members mostly appreciated (health education materials, person who presented, the way it was presented, incentives for their participation)?
- Do you feel like all community members have equal access to the information provided by the education packages?

5. Benefits

We would like to ask your opinion on the benefits of following this education program EPI & ANC.

5A. What are the benefits of conducting the education packages?

Probes:

- What do you think are the benefits of having conducted the education on EPI & ANC?
  - o What are or have been motivating factors for you with the expansion of your role?
- Can you reflect on the application of health education?
- Have you noticed any changes after receiving the information?

5B. What are, in your eyes, the benefits for the community members?

Probes:

- What do you think are the benefits of having conducted the education on EPI & ANC for your community?
  - o What are or have been motivating factors for you with the expansion of your role?
- Where and how has it helped the community to fight off the diseases?
- Have you noticed any changes within the community after receiving the information?

6. Challenges

We would like to ask your opinion on the drawbacks of following this education program EPI & ANC.

6A. What are some challenges of participating in the healthcare education packages?

Probes:

- Encourage a reflection on specific challenges for the current expansion of VMWs
  - Mention specific issues such as transportation, availability of tools to educate community members or motivation (of patient and VMWs themselves).
- Do you ever feel like the current roles and responsibilities are burdensome?

6B. Do you sometimes feel limited in your new role?

Probes:

- Are you missing support to help the community members with the education you have received?
- Any health challenges you see in the community that is not being addressed in one of our four topics (Disease Management, Disease Surveillance, Hygiene & Sanitation or EPI & ANC)?

7. Conclusion

Thank you very much. Would you have anything do add? Do you have any questions?
